# Targeting DNA topoisomerase IIα (TOP2A) in the hypoxic tumour microenvironment using unidirectional hypoxia‐activated prodrugs (uHAPs)

**DOI:** 10.1002/iub.2619

**Published:** 2022-05-02

**Authors:** Paul J. Smith, Stephanie R. McKeown, Laurence H. Patterson

**Affiliations:** ^1^ Cancer and Genetics Division, School of Medicine Cardiff University Cardiff UK; ^2^ Biomedical Sciences Research Institute Ulster University Coleraine UK; ^3^ Institute of Cancer Therapeutics, School of Pharmacy and Medical Sciences, Faculty of Life Sciences University of Bradford Bradford UK

## Abstract

The hypoxic tumour microenvironment (hTME), arising from inadequate and chaotic vascularity, can present a major obstacle for the treatment of solid tumours. Hypoxic tumour cells compromise responses to treatment since they can generate resistance to radiotherapy, chemotherapy and immunotherapy. The hTME impairs the delivery of a range of anti‐cancer drugs, creates routes for metastasis and exerts selection pressures for aggressive phenotypes; these changes potentially occur within an immunosuppressed environment. Therapeutic strategies aimed at the hTME include targeting the molecular changes associated with hypoxia. An alternative approach is to exploit the prevailing lack of oxygen as a principle for the selective activation of prodrugs to target cellular components within the hTME. This review focuses on the design concepts and rationale for the use of unidirectional Hypoxia‐Activated Prodrugs (uHAPs) to target the hTME as exemplified by the uHAPs AQ4N and OCT1002. These agents undergo irreversible reduction in a hypoxic environment to active forms that target DNA topoisomerase IIα (TOP2A). This nuclear enzyme is essential for cell division and is a recognised chemotherapeutic target. An activated uHAP interacts with the enzyme‐DNA complex to induce DNA damage, cell cycle arrest and tumour cell death. uHAPs are designed to overcome the shortcomings of conventional HAPs and offer unique pharmacodynamic properties for effective targeting of TOP2A in the hTME. uHAP therapy in combination with standard of care treatments has the potential to enhance outcomes by co‐addressing the therapeutic challenge presented by the hTME.

**Abbreviation**
AQ4N: Banoxantrone dihydrochloride
BBB: blood brain barrier
CA9: carbonic anhydrase IX
CTCs: circulating tumour cells
CYP450: cytochrome P450
DSB: DNA double strand break
ECM: extracellular matrix
GLUT1: glucose transporter 1 also known as solute carrier family 2, facilitated glucose transporter member 1 (SLC2A1)
GBM: glioblastoma multiforme
HAP: hypoxia‐activated prodrug
HIF: hypoxia‐inducible factor
hTME: hypoxic tumour microenvironment
IQR: interquartile range
MTX: mitoxantrone
PDAC: pancreatic ductal adenocarcinoma
PO2: partial pressure of oxygen
PTEN: phosphatase and tensin homolog
RUNX2: Runt domain transcription factor
TAM: tumour‐associated macrophages
TIFP: tumour interstitial fluid pressure
TOP2: DNA topoisomerase II (EC 5.99.1.3)
TOP2A: DNA topoisomerase IIa
TOP2B: DNA topoisomerase IIb
uHAP: unidirectional hypoxia‐activated prodrug
VEGF: vascular endothelial growth factor

## 1 INTRODUCTION

The tumour microenvironment (TME) comprises regions with very low levels of oxygen (<1%). Such hypoxic TMEs (hTMEs) arise as a result of continued tumour cell proliferation and accompanying vascular insufficiency across a wide range of cancer types.^1^ The hTME can present a key component of clinical resistance to treatment.^2^ The target of hypoxic tumour cells attempts to improve radiotherapy and chemotherapy^3^, ^4^, ^5^, ^6^, ^7^ and immunotherapy.^8^ It has been noted that whilst advances in scoping the complexity of the tumour genome and assessments by immune profiling can offer potential therapeutic strategies, there is a stronger evidence base for directing radiotherapy regimens according to the hypoxic status of an individual's tumour.^9^ Therapeutic options for dealing with tumour hypoxia^10^, ^11^, ^12^ include reversing the low oxygenation of the tumour as a prelude to treatment, interfering with the process of immune evasion, imposing anti‐angiogenic stress to drive tumour fractions to anoxia and necrosis, blocking the molecular pathways for hypoxia sensing or its downstream effectors and overcoming restrictions to drug delivery or activity in the hTME. An attractive option has been to target the hTME using hypoxia‐activated prodrugs [HAPs]. This approach is underpinned by an existing rationale for the use of HAPs as reactive oxygen mimics in chemoradiotherapy.^13^ However, typically, these HAPs are reduced using a 1‐e step which is capable of being reversed; this property can compromise their efficacy and can lead to additional unwanted toxicities.^14^

In this review, we focus on the unidirectional HAPs (uHAPs) AQ4N (banoxantrone) and OCT1002. These uHAPs are both di‐N‐oxide analogues of the anti‐cancer TOP2A poison mitoxantrone.^15^ Activation by a 2‐e reduction step under hypoxia enables the formation of a potent cytotoxin able to trap the nuclear enzyme TOP2A on nuclear DNA. TOP2A is engaged in monitoring and resolving chromosome catenation status prior to cell division^16^ and trapping of the enzyme elicits cell cycle arrest and cell death. This mechanism of action, as a TOP2A poison, is shared by several clinically relevant chemotherapeutic drugs (for extensive reviews see, for example^17^, ^18^, ^19^). However, in their active forms, TOP2‐targeting agents show poor delivery to the hTME due to physicochemical barriers and poor perfusion. This denial of access to a well‐defined hTME can be overcome to varying degrees by uHAP design. The review outlines the characteristics of the hTME and how the uHAP activation mechanism speaks directly to the precision targeting of the hTME whilst minimising normal tissue toxicity. The interaction of an activated uHAP with TOP2A and the cytotoxic consequences are set within the concept of a dynamic hTME. uHAP regimens in combination with agents primarily active in non‐hTME regions have the potential to enhance clinical outcomes by co‐addressing the therapeutic challenge presented by hypoxic tumour fractions in a wide range of solid tumours.

## 2 THE HYPOXIC TUMOUR MICROENVIRONMENT

### 2.1 Defining hypoxia in tumours

**FIGURE 1:**
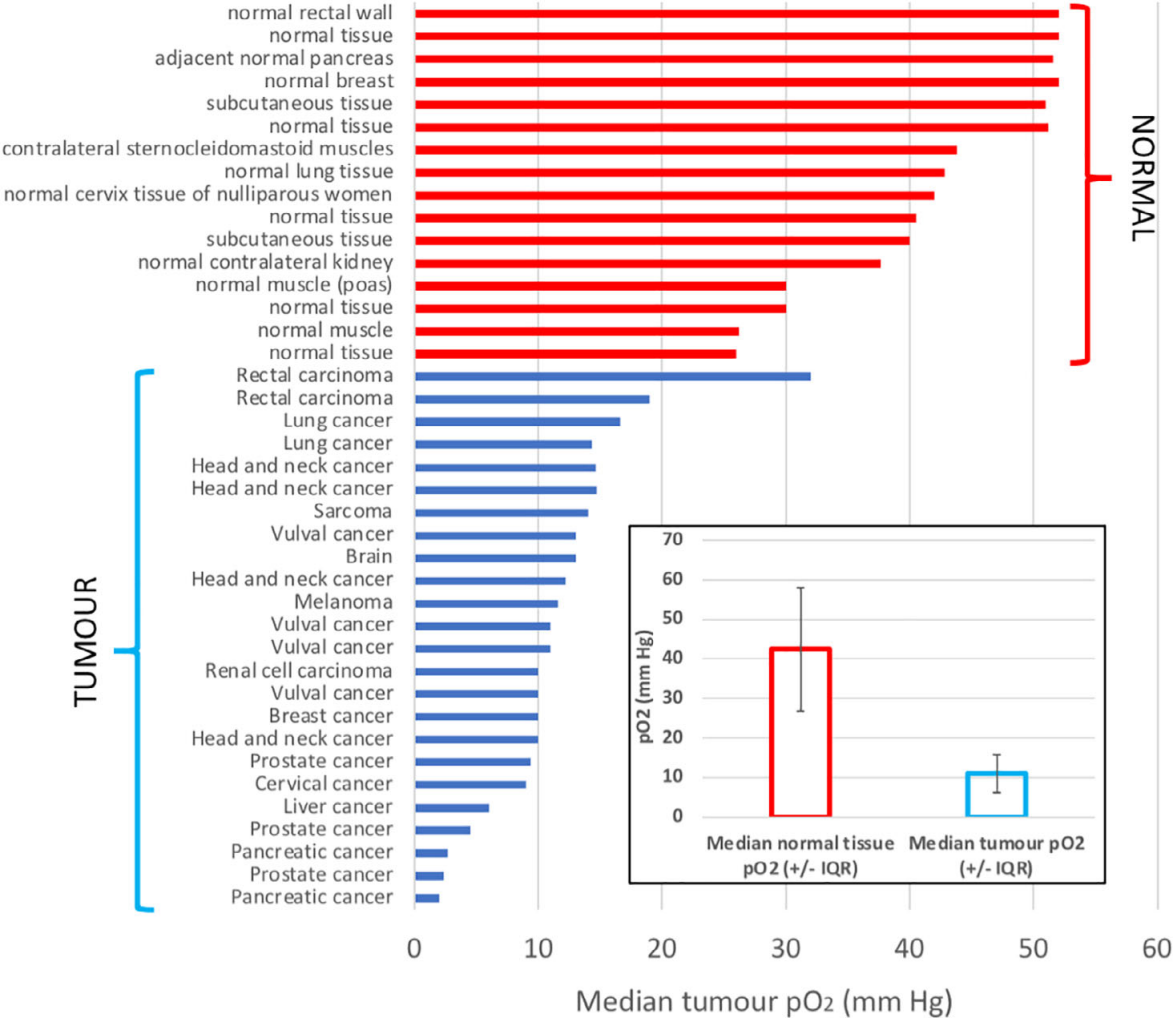
Ranked normal versus tumour oxygenation levels. Much lower levels of oxygen are frequently found in tumour tissues compared to corresponding normal tissue. Where a specific descriptor is given for the normal tissue then this is indicated. Inset shows median values and interquartile range (IQR) for each ranked data set. The range for pO2 (mm Hg): 2.0–32.0 and 26.0–51.6 for tumour and normal tissues, respectively. This range for pO2 nominally corresponds to a range for % oxygen of: 0.3–4.2 and 3.4–6.8 for tumour and normal tissues, respectively. For example, prostate, pancreatic and brain cancers report median values of <2% O2 (<15.3 mm Hg)

In many studies, the level of oxygenation and extent of hypoxia in tumours is poorly understood. It is crucial to recognise that normal tissues have a median pO_2_ level of about 42.6 mmHg (equivalent to 5.6% oxygen)—defined as physoxia. This is much less than inspired air (pO_2 ~_ 176 mmHg; 21% oxygen) which is frequently referred to as 'normoxia'. Although it is difficult to be exact, tumour hypoxia can be considered to occur below 3% oxygen, and in many situations, these levels are significantly lower (<1%). The ability to target the hTME via prodrug activation is a key design feature of HAPs and uHAPs; activation in the hypoxic range thereby protects normal tissues from exposure to the reduction product. In part, HAPs have been compromised during their clinical evaluation by an inability or failure to assess the degree of hypoxia in individual tumours as a means of effective patient selection.^10^ A range of methods are available for evaluation of the extent and degree of tumour hypoxia, but each has limitations prompting a drive towards the development of more accurate imaging approaches translatable to clinical settings.^20^ The pan‐cancer prevalence of tumour hypoxia, based on mRNA abundance signatures, has been reported for 1,188 independent tumours spanning 27 different cancer types.^21^ Using this approach, subsets of patients from 23/27 cancer types were found to have tumours with elevated hypoxia scores, whilst tumours consistently expressed elevated scores compared to normal tissues.^21^ Figure 1 shows a summary, extracting data collated in review,^1^ representing the detection and characterisation of tumour hypoxia as measured using oxygen electrodes in situ. This supports the proposition that almost always the hypoxic status of solid tumours is beyond the normal tissue range of physoxia.

Differentiation between physoxia and tumour hypoxia^1^ is critical for the targeting of the hTME and an expectation of benefit for individual patients and specific tumour types.^22^, ^23^ A recent review of oxygenation in human physiology has clearly described the range of oxygen levels from physoxia through hypoxia to necrosis generating anoxia.^24^ Further, it is important to appreciate that 'normoxia' does not readily replicate *in vivo* tissue physoxic environments. This caveat also applies to preparatory protocols for cell‐based therapies^25^ and in the drug development pipeline where increasingly popular 3D multicellular models demand robust reporters of hypoxic status.^26^ Such a distinction is also important in replicating relevant conditions in pre‐clinical cancer models.^22^, ^27^, ^28^, ^29^

### 2.2 The consequences of hypoxia in the TME

**FIGURE 2:**
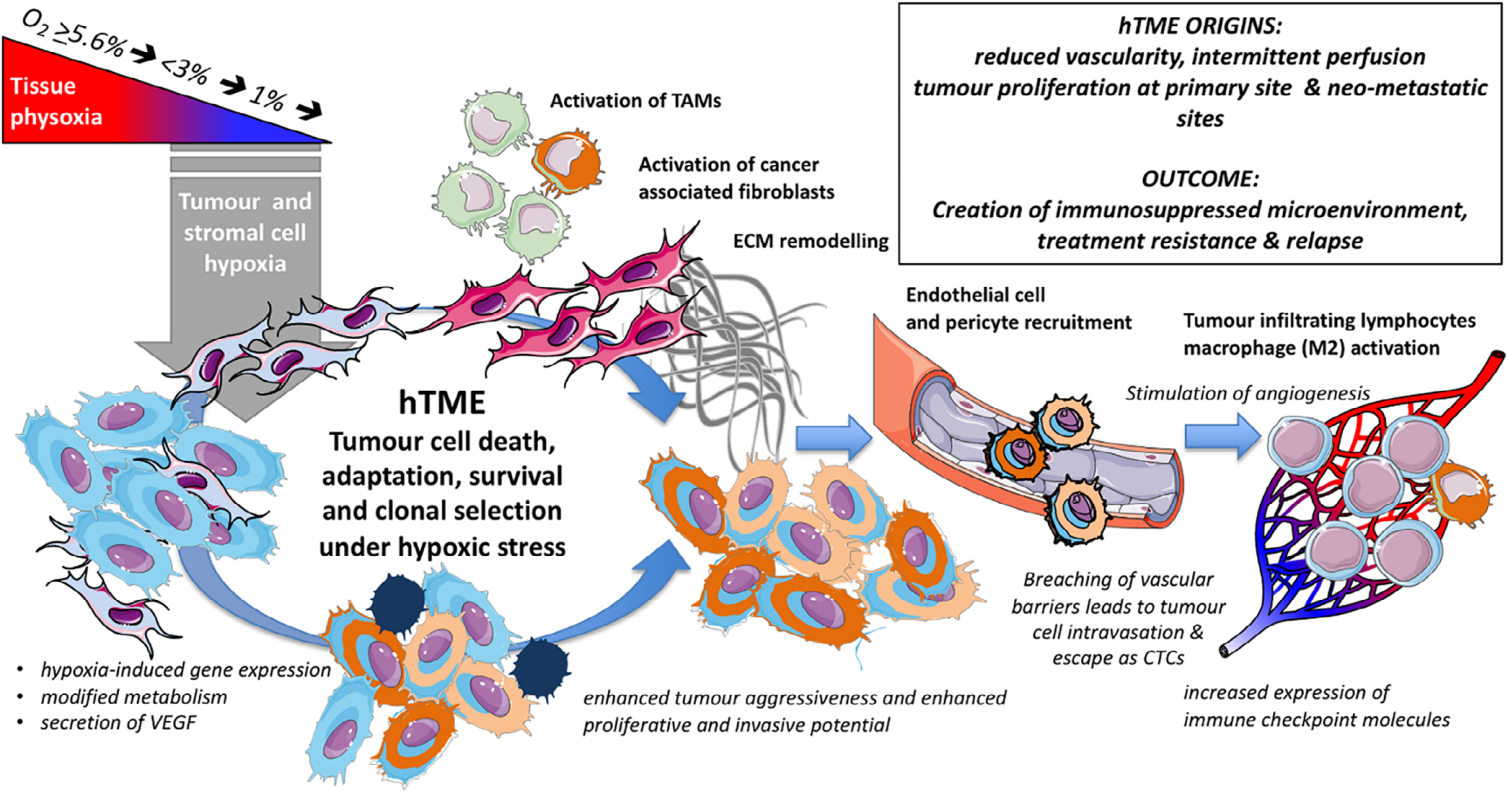
The Hypoxia‐induced Cellular Recruitment Cascade. A series of sequential and parallel events reshape cellular behaviour in the hTME resulting in functional immunosuppression, enhanced tumour aggressiveness, therapeutic resistance and metastasis

Tumour hypoxia drives a plethora of changes that re‐shape cellular behaviour in the hTME, resulting in functional immunosuppression, enhanced tumour aggressiveness, therapeutic resistance and opportunities for metastasis (Figure 2). These changes are mediated by multiple mechanisms including gene expression modifications, oncogene activation and clonal selection. Hypoxia signalling pathways influence the critical steps within this cellular cascade^30^ and key hypoxia response pathways can have particular relevance in a specific cancer (e.g., prostate cancer^31^;). The wider cellular response to hypoxia is primarily orchestrated by the hypoxia‐inducible factor (HIF) family of three heterodimeric transcription factors.^32^, ^33^ The three HIFα subunits (HIF1α, EPAS1/HIF2α and HIF3α) partner with two HIFβ partners (ARNT1 and 2).^12^ The HIF α subunits are oxygen‐labile.^12^ The presence of multiple subunits and partnerships contributes to the versatility that typifies the adaptive responses to the hTME. Under normoxia and sustained expression, HIF1α is rapidly degraded by the ubiquitin‐proteasome system.^34^ However, in an oxygen‐free environment, destruction of HIF1α is blocked and a rapidly mounting level of this subunit in the cytoplasm leads to its translocation to the nucleus and the formation of a heterodimer with its HIFβ partner. The heterodimer HIF1α/HIFβ is then capable of binding to the hypoxia‐responsive elements in target genes.^32^, ^33^, ^34^ Thus, under hypoxia, HIF1α enables the upregulation of genes involved in cancer progression and an adaptive reprogramming of cell metabolism by increasing the abundance of most glycolytic enzymes.^12^

The genetic instabilities of tumour cells in the hTME are subject to Darwinian selection under varying hypoxic stress conditions with downstream impact on DNA repair pathways.^35^, ^36^ This environment drives clonal evolution towards therapy‐resistant phenotypes.^37^, ^38^ Hypoxia has pro‐angiogenic influences on cells within the hTME (Figure 2) and creates routes for the metastatic spread of tumour cells already primed for survival. Such tumour cells are essentially pre‐adapted to hypoxic conditions, potentially aiding the future occupation of microniches distant from the primary site.^39^ An insight into this process is provided by the properties of circulating tumour cells (CTCs) and the role of hypoxia.^40^ A recent report indicates that hypoxia drives intravasation and circulation of tumour cell clusters that appear to retain their hypoxic phenotype.^41^ The Donato et al.'s study^41^ provides evidence that CTC clusters are indeed undergoing hypoxia, whilst single cells show a 'normoxic' signature. The impact of VEGF targeting on the primary tumour can be to increase intra‐tumour hypoxia, resulting in a higher shedding of CTC clusters and metastasis formation.^41^ Consistent with this view is that HIF1α appears to be constantly expressed in large CTC clusters.^42^ In summary, this dynamic environment provides a crucial source of resistance, spread and relapse, which can circumvent or even be aggravated by initial therapy, thereby compromising second‐line treatment.^40^, ^43^ This further underlines the need to specifically target hypoxic tumour cells with uHAP therapy.

Gene expression and mutation profiles of tumours have recently provided evidence that hypoxia can direct the evolutionary trajectory of mutations presented in a given tumour.^21^ The expression/gene signature of a tumour can betray the presence or history of an influence of hypoxia on the tumour presented for treatment. Recent findings in prostate cancer show that the combination of hypoxic stress and tumour cell genomic instability can result in an adverse prognosis.^31^ This is thought to reflect a situation in which hypoxia co‐operates with oncogenic drivers (e.g., loss of PTEN) and suppressing DNA repair capacity with the outcome of changing clonal evolution due to an aggressive mutator phenotype. Further studies and clinical evaluation have the potential for HAPs and uHAPs to demonstrate pan‐cancer activities. Such diseases include prostate cancer progressing to castrate‐resistant forms and diseases with limited treatment options such as pancreatic ductal adenocarcinoma (PDAC) and brain cancers.

Given that the hypoxia‐induced cellular recruitment cascade involves components of immune response pathways (for linked reviews see^44^, ^45^, ^46^:), there are implications for resistance to immunotherapy.^47^ The immunosuppressive impact of the hTME in multiple tumour types is a rapidly expanding field of interest, beyond the scope of the current article, and readers are directed to recent reviews.^3^, ^45^, ^46^, ^48^, ^49^, ^50^, ^51^, ^52^, ^53^, ^54^ In terms of the current review, there is increasing recognition of the hTME as a therapeutic target for the improvement of immunotherapy^50^ and in the design of combination regimens that include HAPs.^46^

### 2.3 Physicochemical barriers within the TME

Coincident with the effects of the very low oxygen levels in the tumour mass, there are physical barriers that change drug access and behaviour. The local extracellular matrix (ECM) comprises multiple components including proteoglycans and fibrous proteins such as the collagens, elastins, fibronectins and laminins.^55^ A recent review^56^ has highlighted the physical traits of cancer which include ECM restructuring and the stromal microarchitecture (Figure 2). A tumour mass often has an elevated tumour interstitial fluid pressure (TIFP). TIFP is particularly important in its effect on tumour blood vessels, due to their deficiency of smooth musculature, increasing the risk of collapse, thereby contributing to both acute and chronic hypoxia. TIFP is a primary barrier for drug delivery to the tumour interior and can have a parallel impact on molecular probes attempting to report on the hypoxic environment itself. Poor lymphatic drainage and chaotic distribution of tumour blood vessels in regions of TIFP also facilitate oxidative stress and acidosis. A reliance on glycolysis for energy production increases hTME acidosis, whilst HIF1α‐dependent induction of carbonic anhydrase IX (CA9) also acts to decrease extracellular pH via increased efflux of carbonic acid.^57^ Such physicochemical factors, including oxidative stress, can combine to compromise drug treatment.^58^

These factors can also affect HAP behaviour within the TME. Prodrug design (reviews^59^, ^60^, ^61^:) attempts to take into account perfusion barriers to tumour penetration. For example, a reduced extracellular pH can impact tumour cell uptake of prodrugs via protonation of basic amino moieties. HAPs incorporating nitrogen mustard alkylating functionality will be more stable in acid environments, possibly favouring their cellular accumulation. Subcellular accumulation may also be determined by the presence of acidic organelles that sequester basic drugs (review^62^:). Further, the mechanism of action of an activated prodrug needs to cope with the modified cellular phenotypes that make up the hTME.^10^, ^14^, ^63^ Even after the successful delivery of an active molecule to a nuclear environment by a DNA‐interactive HAP, for example, chromatin networks^64^ can direct or limit drug access to specific sequences or the sites of functional complexes.^65^ The mechanism of HAP activation is critical and needs to be selective for the hTME.^12^, ^14^ Here, a consideration of 'reversible' HAPs and uHAPs is informative. uHAPs have a major advantage, since the weak charge on the prodrug allows efficient solubility in aqueous environments, whilst also allowing for ease of transfer across membranes providing for a very high volume of distribution even within the hTME.

## 3 PRODRUG ACTIVATION: HAPS AND UHAPS

A prodrug is ideally a pharmacologically inert compound that undergoes biotransformation to facilitate its therapeutic action. Prodrug structures carry non‐toxic functionalities that serve to modify or reduce unwanted properties of the activated compound. This aspect is important for HAP performance to minimise normal tissue toxicity and the dose restrictions typically imposed in combination regimens. An ideal anti‐cancer HAP would undergo intracellular conversion selectively in the hTME^66^ and here, a differentiation of HAPs on the basis of the prevalent mechanism of reduction is informative. A summary of the comparison of the uHAPs with examples of other HAPS and their key mechanisms of action is provided in Table 1.

**TABLE 1:** Comparison of AQ4N and OCT1002 with bioreductive agents

Agent	Chemical class	Mechanism of action	Activating enzyme	Active metabolite
Unidirectional HAPs (uHAPs)^a^				
AQ4N *(Banoxantrone)* & OCT1002	Alkylaminoanthraquinone‐N‐oxide	Topoisomerase II poison	Haemoproteins e.g., CYP's, NOS	AQ4/OCT1001 stable DNA‐affinic agent, oxygen insensitive
Reversible HAPs^a^				
Tirapazamine & SN30000	Benzotriazole di‐N‐oxide	DNA double‐strand breaks	POR	Reactive nitroxyl free radical, redox cycles in air
TH‐302 *(Evofosfamide)*	2‐nitroimidazole‐phosphoramidate mustard	DNA alkylation/cross‐linking	POR	Covalent adduct; redox cycles in air
PR‐104 & CP‐506^c^	Nitroaniline‐nitrogen mustard	DNA alkylation/cross‐linking	POR & AKR1C^b^	Covalent adduct; redox cycles in air

Abbreviations: AKR1C, aldo‐keto reductase; CYP, cytochrome P450; NOS, nitric oxide synthase; POR, cytochrome P450 reductase.

^a^ (Reviews^12^, ^14^, ^67^, ^72^, ^152^, ^153^).

^b^ PR104 but not CP‐506.

^c^ In preclinical development.

### 3.1 Reversible HAPs

Since 1960s, a concept was pursued of harnessing nitroaryl compounds to substitute for reactive oxygen in the radiotherapy of cancers whose treatment was compromised by refractory hypoxia. This gave way to the development of prodrugs bioreductively activated in low oxygen. The view that HAPs undergoing 1e‐bioreduction will generate tumour cell DNA damage via nitro radical anion formation selectively under hypoxia was compelling. Ensuing clinical trials of nitroaryl HAPs to date have largely explored agents with nitroimidazole (TH‐302), nitroaniline (PR104A) or aryl‐di‐N‐oxides (Tirapazamine) bioreduction triggers. TH‐302 has provided the most recent clinical experience for hTME targeting. However, TH‐302 failed to improve overall survival compared with doxorubicin alone in a phase III study of soft‐tissue sarcoma, although several explanations have been proffered following retrospective analyses.^67^ Further, TH‐302 recorded a 'near miss' of the primary end point in another phase III clinical trial evaluating the combination of TH‐302 and gemcitabine in PDAC patients. The experience in PDAC has highlighted the need for patient selection on the basis of the hypoxic status of the tumour,^68^ especially important given the heterogeneity in the levels of hypoxia presented in PDAC.^69^

**FIGURE 3:**
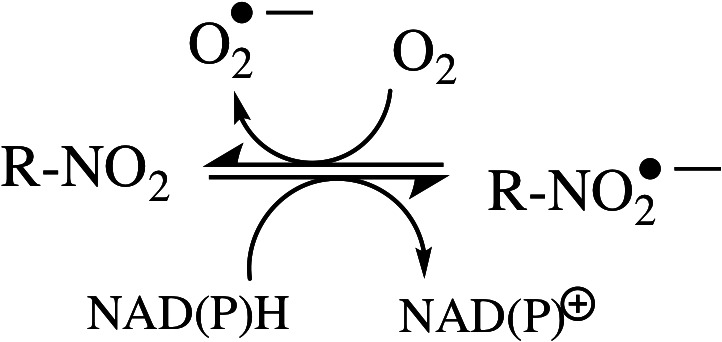
Reactive oxygen formation (superoxide radical anion) and NAD(P)H consumption by nitroaryl HAPs undergoing redox cycling in presence of oxygen

Collectively reversible HAPs rely on a 1e‐bioreduction step, most often catalysed by flavoprotein reductases, a process that can be inhibited even in the presence of moderate hypoxia (typically <1‐5 mm Hg) forcing the HAP to undergo redox cycling (see Figure 3). This diminishes the tumour selectivity of the HAP since normal tissue can also be moderately hypoxic.^1^ In addition, the redox cycling results in reactive oxygen species (ROS), which include superoxide radical anions and hydroxyl radicals, are well known to be toxic to normal tissues.^70^ Further, biological redox cycling in an aerobic tissue by one molecule of a reversible HAP will give rise to appreciable levels of a variety of ROS. This is exacerbated by additional oxidative stress through depletion of cellular NAD(P)H used as reducing equivalents by the HAP‐activating flavoprotein reductases. The other disadvantage of a reversible HAP is, in principle, that induced DNA damage is confined to the cell the HAP is activated in. This diminishes the opportunity for a prolonged anti‐tumour effect, whereby the activated HAP could maintain its cytotoxicity whatever the prevailing cellular oxygen tension or of a bystander effect against cells located close to the hypoxic niches within the tumour.

A more recent approach for HAP design is to incorporate a standard bioreductive trigger to release a pharmacological agent that interferes with a specific cellular process, and these include: the experimental agent CH‐01 that releases a Chk1/Aurora A inhibitor following nitroimidazole bioreduction^71^; BCCA621C that releases a DNA‐dependent protein kinase inhibitor following nitroaniline bioreduction^72^; NBGNU, a hypoxia‐activated combi‐nitrosourea prodrug designed to release an inhibitor of *O* ^6^‐alkylguanine‐DNA alkyltransferase (AGT); a type I DNA topoisomerase targeting HAP involving C‐10 substituted derivatives (2‐, 3‐ and 4‐nitrobenzyl) of a camptothecin analogue (SN‐38).^73^

### 3.2 Unidirectional HAPs

HAP design employing aliphatic N‐oxides, that can be activated through an obligate 2e‐bioreduction process, addresses the disadvantages of reversible HAPs. A key characteristic of the activation process is that the intrinsic oxygen atom transfer under hypoxic conditions is a unidirectional reaction resulting in *irreversible* formation of a *stable* targeted agent and water as a by‐product.^15^ The reduction product will remain localised to the hTME for some considerable time and therefore has the capability to kill both the hypoxic cells in which it is formed and to exert a bystander effect on others located in the immediate locality.^74^

### 3.3 AQ4N

**FIGURE 4:**
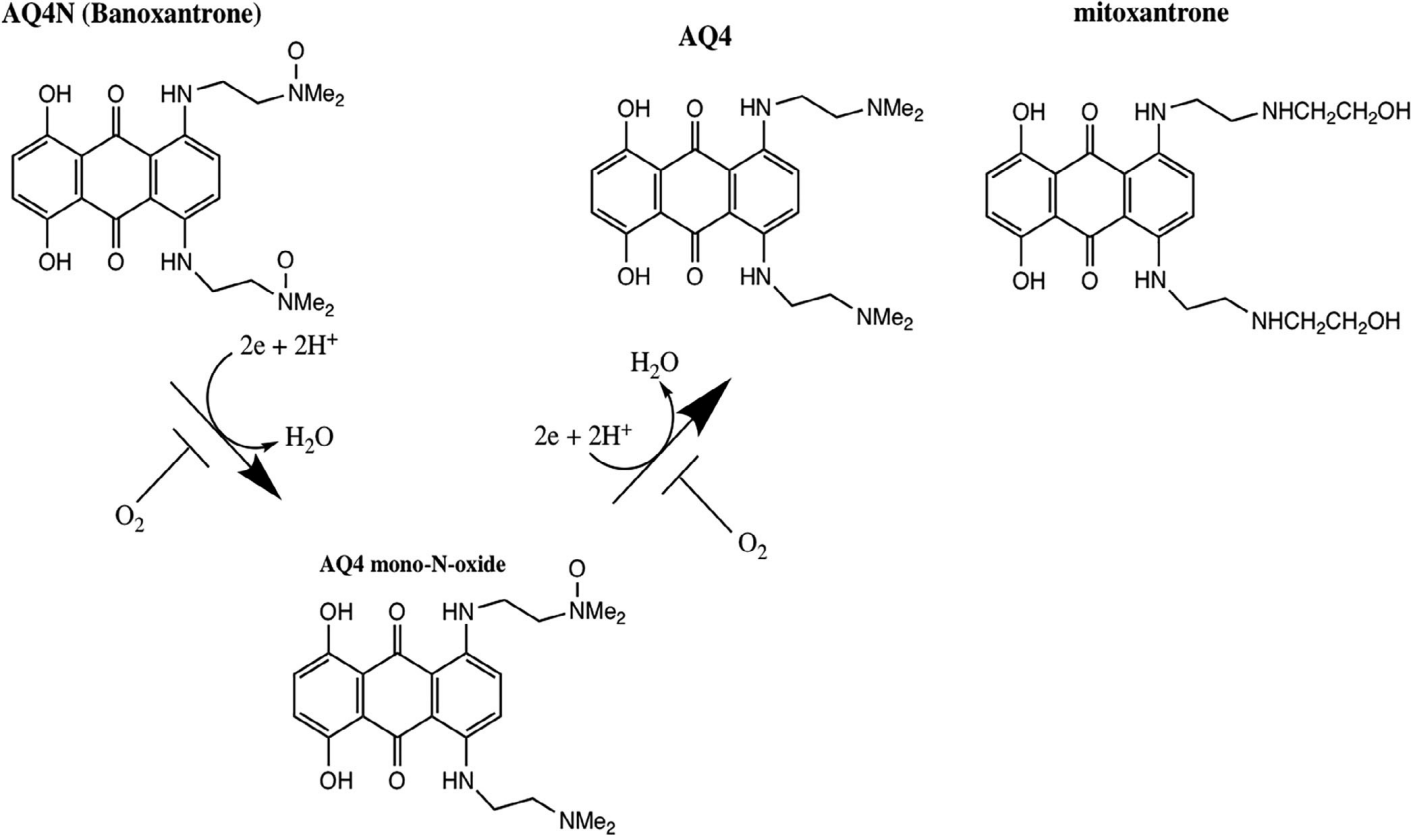
Activation process for the uHAP AQ4N under hypoxic conditions

The first compound of the uHAP type to be developed was AQ4N.^75^, ^76^ Under hypoxia, the drug undergoes two sequential 2e—reductions, via the mono N‐oxide AQ4M, to give the toxic metabolite AQ4—a metabolically stable reduction product with high affinity for its selected targets. AQ4N is metabolised by haemoproteins acting as the 2e‐reductases (Figure 4), but only in the absence of oxygen. Haemoproteins include CYP450 isoforms, nitric oxide synthase, haem oxygenase, deoxyferrohaemoglobin and degraded ferroprotein.^15^, ^75^ The cytochrome P450 (CYP450) superfamily is a case in point and has featured significantly in judging the potential for AQ4N activation given that epigenetic mechanisms can modulate the expression of CYPs over a human's lifespan, in diverse organs^77^ and also under hypoxia.^78^ Specific CYP isoforms CYP2S1 and CYP2W1, capable of reducing AQ4N, have also been reported.^79^

Insight into the action of the activated form of AQ4N can be found in its structural similarity to mitoxantrone (MTX), an anti‐cancer DNA intercalator and TOP2A poison (see Figure 4). MTX is the leading example of a group of anti‐cancer anthracenedione compounds that include ametantrone and pixantrone. MTX, like the anthracycline doxorubicin, shows evidence of multi‐level action^80^ under non‐hypoxic conditions,^81^ including a chromatin disruption effect of DNA adduct formation. However, MTX, unlike doxorubicin, does not engage in cell‐damaging redox activity and lipid peroxidation. MTX can also act as a HIF1α inhibitor^82^ and shows low nanomolar inhibitory activity against PIM1 kinase.^83^ PIM1 kinase has enhanced activity in the hTME,^84^ promoting resistance to anti‐angiogenic agents.^85^ The kinase is linked to malignancy in a range of cancers^86^, ^87^, ^88^, ^89^, ^90^ including glioblastoma multiforme (GBM)^91^, ^92^ and is now a recognised therapeutic target.^93^ These properties of MTX indicate the potential of the pharmacophore to provide a variety of desirable anti‐tumour properties, which can be used to design a 'multi‐action prodrug'^94^ to target the hTME.

Consequently, MTX is an informative prototype pharmacophore for the development of the di‐N‐oxide uHAPs.^15^, ^95^, ^96^ The lead compound, AQ4N, was designed by selective modification of the 1,4 disubstituted alkylamino side chains important for the stabilisation of DNA binding. The modification comprises a neutralisation of the cationic charge on MTX, creating a prodrug which is susceptible to irreversible reduction to AQ4 — the active chemotherapeutic agent in hypoxic environments (Figure 4). The 2‐e reduction step avoids the possibility of redox cycling if cells become better oxygenated.^15^, ^97^ Crucially, charge neutralisation of the prodrug also significantly increases the capacity for tumour mass penetration, including the hTME; access well beyond that observed with MTX.^98^ In essence, AQ4N acts as a 'non‐toxic' delivery agent for the potent MTX‐like toxin, AQ4, which remains within the hTME for many hours or even days due to its high affinity for DNA.^96^ Charge neutralisation of the pharmacophore also significantly lessens the risk of adverse systemic effects including cardiotoxicity which is associated both with the anthracyclines and MTX.^99^

These design assets are supported by an extensive literature on the anti‐cancer properties of AQ4N including safety and low toxicity in several clinical studies.^100^, ^101^, ^102^, ^103^, ^104^, ^105^, ^106^ Multiple preclinical studies have highlighted the potential of AQ4N to enhance significantly both radiotherapy^107^, ^108^, ^109^, ^110^, ^111^ and chemotherapy^76^, ^108^, ^112^, ^113^, ^114^ since AQ4N provides the ability to target hypoxic cells which are significantly resistant to these modalities. In addition, AQ4N has been shown to inhibit both deleterious genetic changes and metastatic spread of prostate tumours in a preclinical model.^115^ The AQ4N activation mechanism has also been integrated into novel combination concepts for photodynamic therapy, further expanding the uHAP principle.^116^

The therapeutic uHAP challenge varies, at least, according to tumour type and stage. A biopsy study has shown that AQ4N is metabolised to AQ4 in a range of different solid tumours including GBM, bladder, head and neck and breast.^100^ This confirms that human tumours in situ are capable of metabolising AQ4N. In addition, tumour sections showed AQ4 accumulation at bioactivity‐relevant levels that colocalise within regions of Glut‐1+ hypoxic cells, suggesting that reduction is related to the level of hypoxia in the TME. The majority of standard therapeutic agents have limited use in the treatment of brain tumours due to their inability to cross the blood–brain barrier (BBB). Proof‐of‐principle of action by the electrically neutral AQ4N beyond the BBB has been obtained for patients with GBM,^100^ opening therapeutic possibilities in the treatment of brain tumours.^117^ To date, AQ4N has not progressed beyond the demonstration of safety in Phase I setting but is primed for an evaluation of efficacy under appropriate trial designs.^68^

### 3.4 OCT1002

This is a second generation selectively deuterated di‐N‐oxide analogue of AQ4N with intrinsic properties described previously.^118^ Deuterium isotope effects on drug pharmacokinetics have been described^119^ and these await evaluation for OCT1002. The retention of the deuterated moieties of the activated drug (OCT1001) has the potential to modify non‐covalent interactions between molecules.^120^, ^121^ Since activation under hypoxia is intracellular, differences in the polarities of deuterated versus non‐deuterated isomers^122^ also offer the prospect for deuterated prodrugs to distribute differentially at subcellular locations and vesicle compartments.

OCT1002 has shown significant anti‐cancer activity in prostate cancer models in which hypoxia has been confirmed,^118^, ^123^ demonstrating growth inhibition and suppression of metastasis. This aligns with earlier *in vivo* studies with AQ4N that have shown anti‐angiogenic^124^, ^125^ and anti‐metastatic activities.^115^, ^126^ These effects were supported by data from PCR arrays which demonstrated that significant gene expression changes occur during 28 days of anti‐androgen (bicalutamide) treatment of LNCaP prostate tumours *in vivo*. These changes were substantially blocked by combination treatment with OCT1002.^118^ In particular, the overexpression of the Runt domain transcription factor (RUNX2) was blocked. RUNX2 is involved in hypoxia‐driven angiogenesis and several of its downstream targets are associated with pro‐survival pathways.^127^ The changes in RUNX2 expression were confirmed using immunohistochemistry to identify the protein product. The proliferation marker Ki67 was similarly inhibited. Further analysis of the data identified an upregulation of many genes following anti‐androgen treatment, especially after 21 days, an effect that was largely absent when OCT1002 was combined with bicalutamide. It was postulated that the profound hypoxia, induced in the early days of treatment (measured using an oxygen electrode), may have been selected for more stress‐resistant, malignant cells, whilst the combination with the uHAP significantly inhibited this process. These outcomes have implications for the design and refinement of existing androgen‐deprivation regimens for prostate cancer in the clinic.^118^

## 4 UHAP TARGETING OF TOP2A IN THE HTME

### 4.1 Target interactions

**FIGURE 5:**
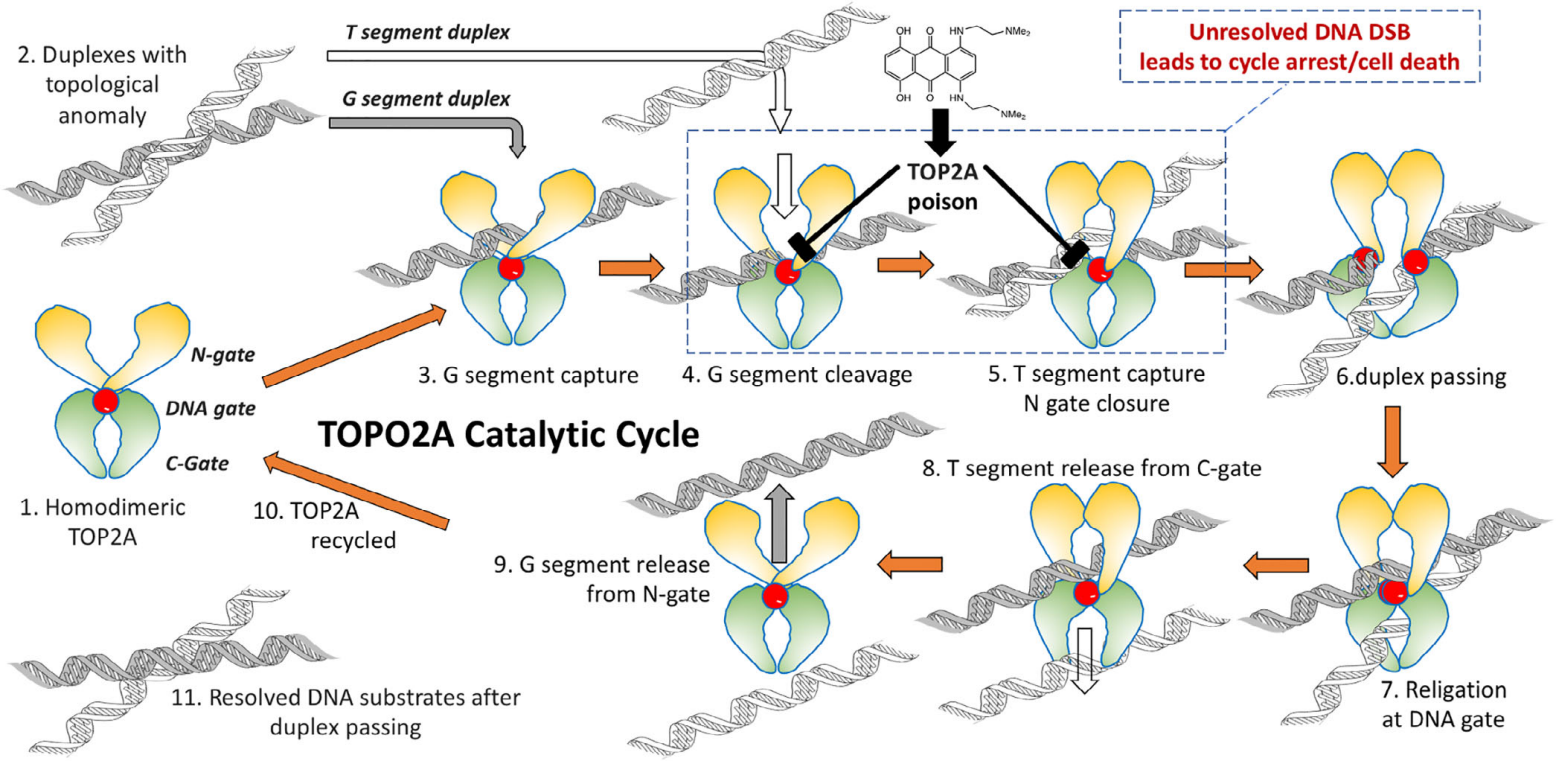
Diagrammatic representation of the catalytic cycle of TOP2A (1) and poison‐induced enzyme trapping at the DNA gate. The pathway shows the resolution of a topological anomaly (2) in DNA that requires the passing of one intact DNA duplex through another. The normal catalytic activity of TOP2A (3–9) creates and reseals a DNA double‐strand break (DSB) via a sequence of steps to allow duplex passing and release permitting the recycling of the enzyme (10) and resolution of the topological anomaly. TOP2A poisons act primarily at step 4, trapping the complex with a sequestered DSB. If the trapped TOP2A cannot be adequately repaired, then tyrosyl‐DNA phosphodiesterase 2 or nuclease will remove the TOP2A from the complex, leading to a persistent and irreversible DNA DSB. A mounting level of DSBs induces cell cycle arrest and subsequent cell death

Chromatin is a store of torsional energy reflecting topological stress or anomalies,^128^ features resolvable by DNA topoisomerases. TOP2A (a type IIA DNA topoisomerase) is a nuclear enzyme which acts as a 'full decatenase',^19^ passing one duplex through a DNA double‐strand break (DSB) generated by the dimeric enzyme in an opposing duplex within the enzyme DNA complex. This feat is achieved, without signalling DNA damage, by each protomer maintaining a covalent phosphotyrosyl linkage to the 5′‐end of the DSB during strand passing followed by religation of the break and enzyme release (Figure 5). The steps in the process are highly co‐ordinated.^129^ The enzyme first associates with the G‐segment duplex and catalytic tyrosines initiate a transesterification reaction acting at staggered phosphodiester bonds on opposite DNA strands. This yields the classical 'TOP2 cleavage complex'—a potentially dangerous construct unless further resolved by key steps: an ATP‐operated gate (the N‐gate) closes to capture and drive the T‐segment through the DNA‐gate; the enzyme becomes available for its next catalytic cycle by releasing the T‐segment through the C‐gate and religation of the cleaved G‐segment by reversal of the transesterification reaction; finally, a re‐opening of the N‐gate upon ATP hydrolysis.^129^ TOP2 poison drugs interact with the closed conformation of the DNA‐gate and inhibit the religation of cleaved G‐segment by the occupation of the DNA cleavage site.^129^, ^130^ The human TOP2 proteins, TOP2A and an isomer TOP2B, are the targets for several anti‐cancer agents^64^, ^131^ including etoposide, intercalating anthracyclines (doxorubicin and daunorubicin) and the anthraquinone MTX.^132^ These drugs act as poisons stalling the enzyme at the 'TOP2 cleavage complex' stage (outlined in Figure 5). This inhibition of strand religation and subsequent attempts at damage processing disrupt cell cycle progression and recruit DNA damage stress responses.^100^, ^133^

An earlier modelling study supported the repulsion of AQ4N from DNA,^134^ a pre‐requisite for the non‐toxic action of this prodrug and excluding any TOP2A poisoning potential. Confocal imaging has revealed significant nuclear exclusion of AQ4N in contrast to nuclear retention of its reduction product AQ4^96^ with subsequent TOP2A trapping.^135^ AQ4 has properties that closely reflect those observed for MTX.^132^, ^136^, ^137^ This includes long‐lived stabilisation of enzyme‐DNA complexes at lower concentrations (t_1/2_ 10 h for TOP2A, t_1/2_ 6 h for TOP2B in mouse embryonic fibroblasts^138^;). The long‐lived trapping of TOP2A and TOP2B ternary complexes well beyond those achieved by the classical TOP2 poisons etoposide and mAMSA^137^ lead to sustained DNA damage induction^139^ and the persistent inhibition of DNA synthesis.^132^

The distinction between the description of the mechanism of action as 'poison or inhibitor' has been addressed in a recent review.^140^ At high MTX concentrations, there is suppression of complex formation. Consequently, such agents have been described as topoisomerase II 'poisons' when acting at low concentrations and 'inhibitors' at high concentrations.^81^ For a high‐affinity DNA ligand (AQ4) generated via hypoxic activation of its low‐affinity uHAP (AQ4N),^96^ pharmacodynamic action will initially be through prolonged complex trapping‐mediated DNA damage—a poison‐mediated route. The high *in vivo* activity of AQ4N^97^ relative to another topoisomerase‐targeted N‐oxide, DACA‐NO, has suggested that the release in hypoxic cells of an intercalator (AQ4) with sufficiently high DNA binding affinity will also provide for drug persistence within hTMEs, as also shown for activated OCT1002.^118^ AQ4N shows increased tumour penetration as compared to other drugs like MTX.^98^

### 4.2 Cell cycle, topoisomerases and the hTME

TOP2A is vital for cell cycle progression^141^ and is downregulated in non‐cycling cells.^142^, ^143^ TOP2B expression shows a significantly reduced dependency on active cell cycle progression and also appears to be non‐essential for cell viability.^137^ TOP2B, therefore, presents a co‐target less affected by proliferation status. Re‐entry of a quiescent tumour cell into the cell cycle results in a spike of TOP2A synthesis prior to both S phase entry and mitosis,^142^ aligning with the need to anticipate the resolution of chromatin topological anomalies at critical points in the cell cycle. Upon re‐entry in the presence of TOP2 poisons, there is increased cross‐linking potential, leading to cell cycle arrest and cell death.^141^ TOP2A amplification is present in a range of solid tumours, known to contain hTMEs. For example, HIF1α can be stabilised through the activation of HER receptor signalling^144^ and in breast cancer there is evidence of high rates (>40%) of HER2 and TOP2A co‐amplification.^145^ TOP2A has also been associated with metastatic activity in forms of pancreatic cancers.^146^, ^147^

**FIGURE 6:**
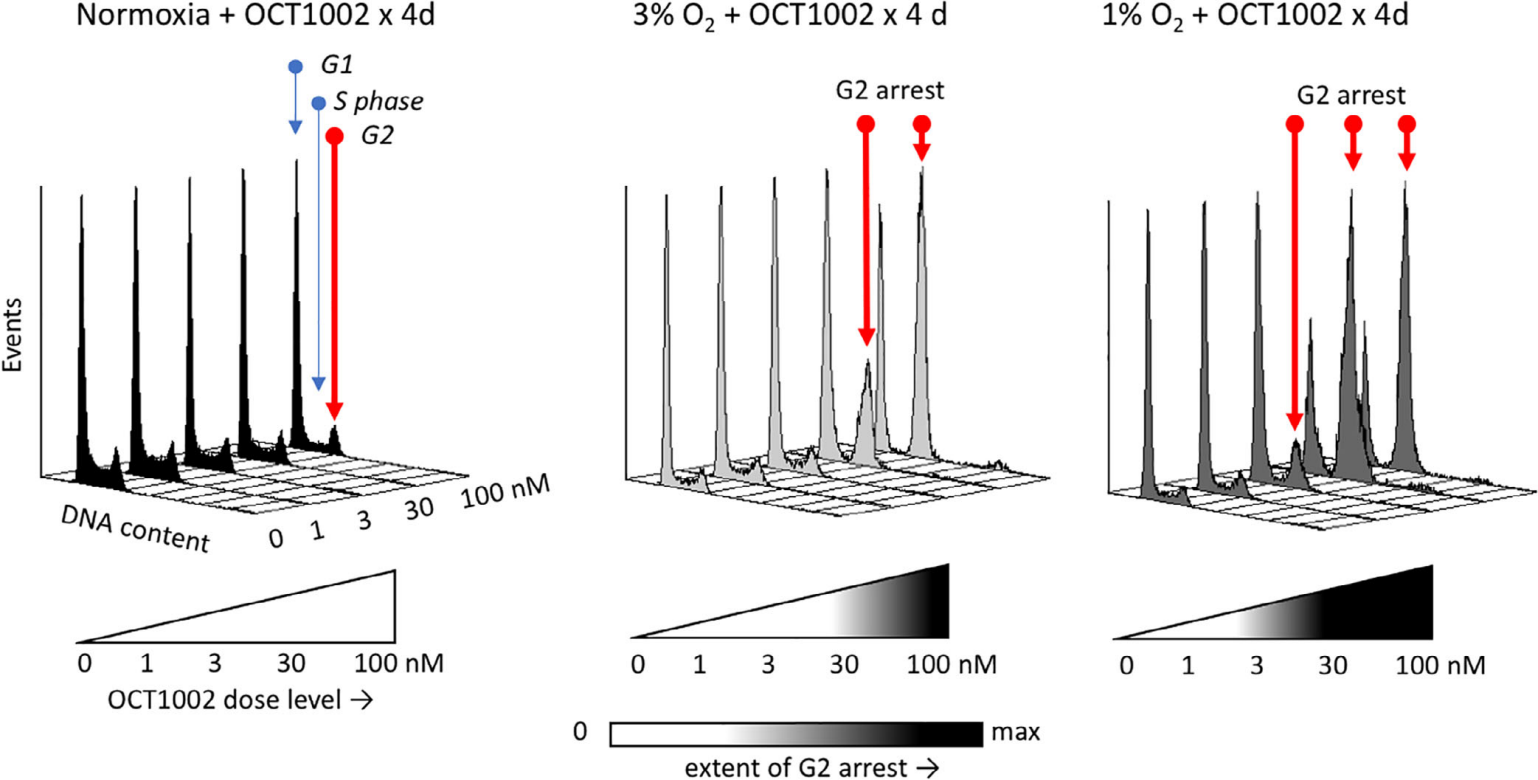
Cell cycle arrest of human A549 cells under *in vitro* co‐exposure to varying oxygen levels and OCT1002. Histograms show the flow cytometric analyses of the distribution of cellular DNA content under given conditions. Under a 4‐day continuous exposure, OCT1002 induces a dose‐dependent late cell cycle (G2) arrest only under hypoxic conditions (arrest at 1% O_2_ > arrest at 3% O_2_)

Cells in the hTME undertaking cell cycle progression face late cell cycle checkpoints activated by failed DNA decatenation or an accumulation of unresolved DNA damage.^16^, ^148^ Figure 6 shows a typical flow cytometric analysis of DNA distributions of human A549 lung adenocarcinoma cells undergoing pre‐mitotic G2 arrest during prodrug exposure under sustained hypoxia. OCT1002 causes both dose‐dependent and hypoxia‐dependent arrests. The normoxic controls (20% oxygen) show no arrest at all drug concentrations, whereas OCT1002 (20–100 nM) under 3% O_2_ demonstrates G2 arrest. This cycle arrest effect is initiated at an even lower dose level (3 nM) under 1% oxygen.

In summary, the key properties of an activated uHAP that targets the hTME are as follows: (i) capacity to be delivered to the hTME,^100^ (ii) the selectivity for activation in hTME without significant systemic toxicity,^100^, ^101^, ^102^, ^103^, ^104^, ^105^, ^106^ (iii) the prolonged residence of activated prodrug at DNA sites due to high affinity for the target,^135^ (iv) the availability of activated drug from sequestered cellular stores,^96^ (v) persistent co‐location of activated uHAP within poorly vascularised tumour regions,^118^ (vi) the targeted long‐lived trapping of TOP2A/TOP2B ternary complexes.^138^

## 5 CONCLUSIONS

There is clearly a de facto case for the continued pursuit of overcoming the adverse impact of tumour hypoxia on treatment options and outcomes. This is further highlighted by the growing need to address underlying reasons for the failure of promising approaches to immunotherapy. This review has attempted to bring a focus on the importance of uHAP design in targeting the hTME and address the restrictions and limited therapeutic usefulness of the reversible HAPs. It is increasingly clear that the impact of the hTME is far‐reaching and not just confined to tumour cells in situ. Such niches recruit local stromal cell cohorts and multiple cell types that both infiltrate and transit the hTME—whether at primary or metastatic sites. Neoplastic cells escaping from primary niches present a tipping point for treatment options and the anti‐metastatic effects of uHAPs are clearly relevant in this scenario.

The review has set uHAP development against a backdrop of the profiles of hypoxia and physoxia found in preclinical tumour models and in a range of the most clinically prevalent cancers (see Figure 1). *In vitro* experimental evaluations should at least attempt to mimic these low oxygen levels and deploy new generations of hypoxia reporters for 3D and organoid culture models. This underlines the problem when trying to re‐create the hTME experimentally and indicates the importance of an existing clinical proof‐of‐concept already gained for AQ4N.^100^ Further, there is a growing consensus on the need for the development of relevant imaging biomarkers, especially for hypoxia.^149^, ^150^, ^151^

Incorporating an understanding of the complexity of the hTME and its impact on cell proliferation either at the primary site or after metastatic spread will also help to inform smart drug combinations that can incorporate uHAPs. We have drawn scenarios of how a multi‐level acting uHAP can have pharmacodynamic effects via both TOP2‐dependent and independent routes. The view of a uHAP as a 'precision' medicine relates to the targeting of the hTME. Treatment is predicated on the principle that activation only occurs in areas of profound hypoxia, a feature rarely found in normal tissues. This principle is supported by the observed limited systemic toxicity of AQ4N in preclinical and clinical studies. The second level of precision relates to the nature and role of the ultimate intracellular drug target within the hTME and in the case of uHAPs reflects the recognised validity of TOP2 targeting. This second level does not preclude the involvement of alternative routes for interfering with cellular hypoxia response pathways.

It is becoming generally accepted that a key step in combatting the deleterious influence of tumour hypoxia on treatment outcomes in patients requires an evaluation of the hypoxic status of individual tumours. This would create a logical route for the selection of patients for a uHAP in combination therapies. The uHAP principle of reducing off‐target action to a minimum provides a rationale for uHAP combination therapies to minimise the role of hypoxia in treatment failure. Under innovative clinical trial design, this could allow for an early introduction of uHAPs into a treatment path with pan‐cancer implications.

## CONFLICT OF INTEREST

PJS (director), SRM (director) and LHP are shareholders of OncoTherics Limited. PJS and LHP are directors and shareholders of BioStatus Limited. BioStatus created OncoTherics Limited to commercially exploit intellectual property concerning a new series of anti‐cancer drugs that can be activated under hypoxia. PJS, SRM and LHP are actively involved in commercialising this technology through OncoTherics Poland S.A.

## AUTHOR CONTRIBUTIONS

Writing, review and/or revision of the manuscript: all authors.
